# The role of Atg16 in autophagy, anthocyanin biosynthesis, and programmed cell death in leaves of the lace plant (*Aponogeton madagascariensis*)

**DOI:** 10.1371/journal.pone.0281668

**Published:** 2023-02-16

**Authors:** Nathan M. Rowarth, Adrian N. Dauphinee, Christian R. Lacroix, Arunika H. L. A. N. Gunawardena

**Affiliations:** 1 Department of Biology, Dalhousie University, Halifax, NS, Canada; 2 Department of Molecular Sciences, Uppsala BioCenter, Linnean Centre for Plant Biology, Swedish University of Agricultural Sciences, Uppsala, SE, Sweden; 3 Department of Biology, University of Prince Edward Island, Charlottetown, PEI, Canada; Universidade do Minho, PORTUGAL

## Abstract

*Aponogeton madagascariensis*, commonly known as the lace plant, produces leaves that form perforations by programmed cell death (PCD). Leaf development is divided into several stages beginning with “pre-perforation” furled leaves enriched with red pigmentation from anthocyanins. The leaf blade is characterized by a series of grids known as areoles bounded by veins. As leaves develop into the “window stage”, anthocyanins recede from the center of the areole towards the vasculature creating a gradient of pigmentation and cell death. Cells in the middle of the areole that lack anthocyanins undergo PCD (PCD cells), while cells that retain anthocyanins (non-PCD cells) maintain homeostasis and persist in the mature leaf. Autophagy has reported roles in survival or PCD promotion across different plant cell types. However, the direct involvement of autophagy in PCD and anthocyanin levels during lace plant leaf development has not been determined. Previous RNA sequencing analysis revealed the upregulation of autophagy-related gene *Atg16* transcripts in pre-perforation and window stage leaves, but how Atg16 affects PCD in lace plant leaf development is unknown. In this study, we investigated the levels of Atg16 in lace plant PCD by treating whole plants with either an autophagy promoter rapamycin or inhibitors concanamycin A (ConA) or wortmannin. Following treatments, window and mature stage leaves were harvested and analyzed using microscopy, spectrophotometry, and western blotting. Western blotting showed significantly higher Atg16 levels in rapamycin-treated window leaves, coupled with lower anthocyanin levels. Wortmannin-treated leaves had significantly lower Atg16 protein and higher anthocyanin levels compared to the control. Mature leaves from rapamycin-treated plants generated significantly fewer perforations compared to control, while wortmannin had the opposite effect. However, ConA treatment did not significantly change Atg16 levels, nor the number of perforations compared to the control, but anthocyanin levels did increase significantly in window leaves. We propose autophagy plays a dual role in promoting cell survival in NPCD cells by maintaining optimal anthocyanin levels and mediating a timely cell death in PCD cells in developing lace plant leaves. How autophagy specifically affects anthocyanin levels remained unexplained.

## Introduction

Programmed cell death (PCD) is a highly controlled cellular process that removes compromised cells from environmentally induced stress or designated cells by developmental regulation to respectively achieve survival or tissue remodeling [1–3]. Common examples of developmental PCD in plants used to achieve higher tissue organization include aerenchyma formation and xylem differentiation, suspensor and tapetum cell deletion, the dismantling of rapidly growing root tip cells, and organ senescence [4–10]. The molecular pathways that control PCD in animal models are relatively well identified compared to those involved in plant developmental PCD which is less understood [5, 11, 12].

The aquatic monocot *Aponogeton madagascariensis* (commonly known as the lace plant) is an emerging model system to investigate plant developmental PCD [13–15]. Leaves of the lace plant form laminar perforations during normal development by PCD [13, 14]. Leaves emerge from the corm in a heteroblastic series, and leaf development has been defined into several stages. The first 3 to 4 “imperforate” leaves that emerge do not form perforations by maturity. Successive leaves form perforations and go through the following stages of development. Pre-perforation leaves emerge furled and the lamina tissue framed by the vasculature (called areoles) is enriched with red pigment from the accumulation of anthocyanins. As leaves develop into the window stage anthocyanin recedes towards the veins and a gradient of red pigment remains. Cells central to the areole are void of pigment and destined for death (PCD cells). Cells undergoing PCD die off from the center of the areole progressively towards the veins up until 4–5 living cell layers are left adjacent to the veins. Cells that retain anthocyanin will eventually lose their red pigmentation during development and maintain homeostasis; designated as non-PCD, NPCD cells [16, 17].

**Fig 1:**
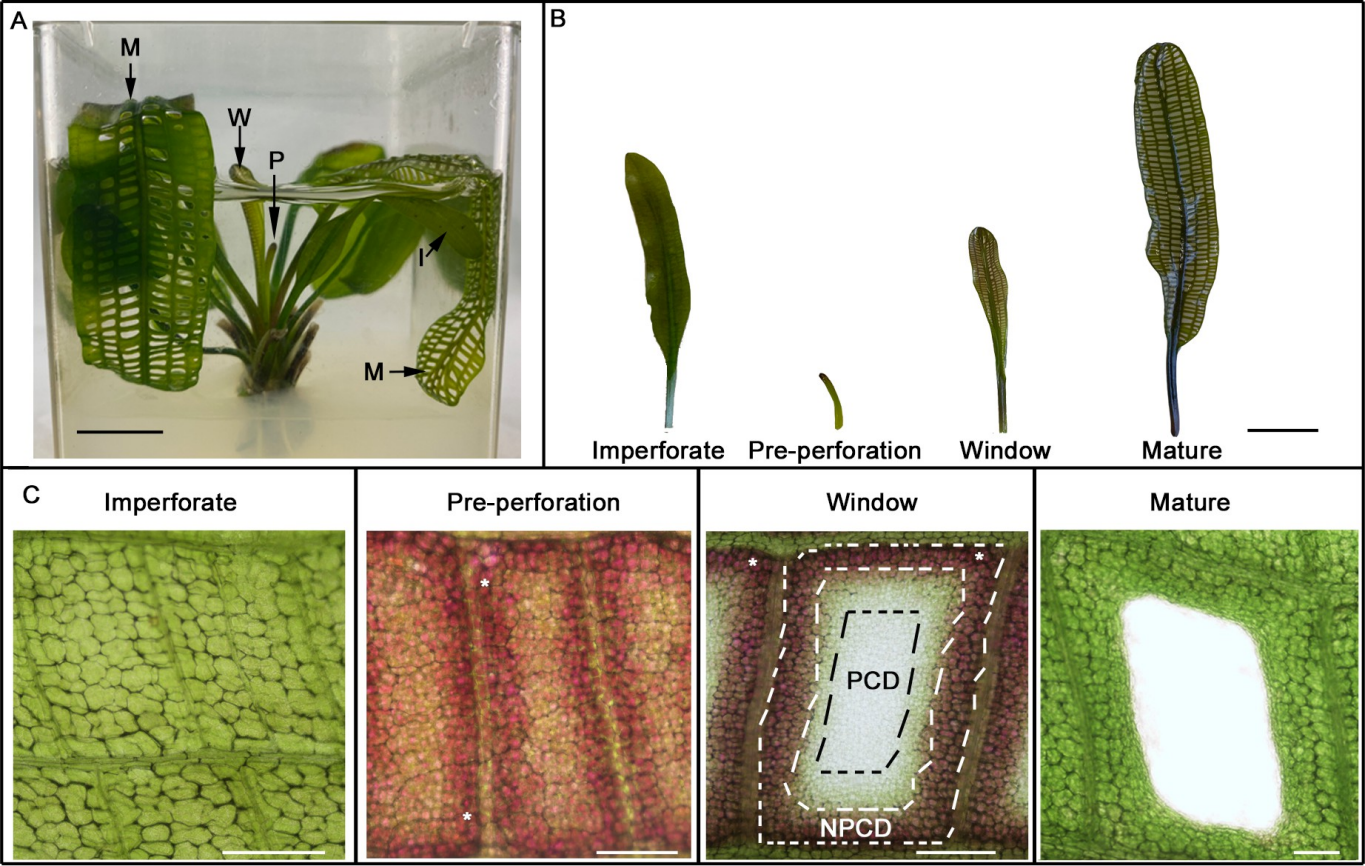
The lace plant programmed cell death (PCD) system. (**A**) Lace plants are grown in axenic Magenta box culture; pre-perforation stage (P), window stage (W), mature stage (M), and imperforate leaves (I). (**B**) Representative leaves at different stages of development. Imperforate leaves are the first 3–4 leaves to emerge from the corm and do not produce perforations. Pre-perforation stage leaves emerge from the corm with anthocyanin pigmentation. These eventually develop visible areolar “windows” that will eventually become perforated in mature leaves. (**C**) Details of PCD process. PCD does not take place in imperforate leaves. In pre-perforation leaves, anthocyanin pigmentation is visible, especially at the periphery of the areole (*asterisks*). PCD can be seen actively occurring in the window stage of development as a gradient of cell death. Non-PCD cells (NPCD, bounded by *white dashed lines*) persist beyond maturity, early-PCD cells (EPCD, bounded by inner *white dashed lines* and *black dashed lines*) have lost their anthocyanins and are destined to die, and late-PCD cells (LPCD, bounded by *black dashed lines*) are nearly transparent and on the verge of death. Perforation formation is completed in mature stage leaves. Anthocyanin abundance is visibly reduced, and homeostasis for NPCD cells is reached. *Scale bars*: A = 1 cm, B = 2 cm, C = 70 μm.

The lace plant is an emerging model system (Fig 1) to study plant PCD due to the predictable nature of the spatiotemporal separation of NPCD and PCD cells within areoles. The natural translucency of lace plant leaves is ideal for live-cell microscopy. Additionally, the established protocol for the propagation of whole lace plants in sterile axenic environments allows for pharmacological studies [13, 14, 18]. The chronological order of intra-cellular events in lace plant areoles during PCD is well characterized [16, 19–23] but the molecular pathways that are critical to lace plant PCD are less well understood.

In many plant systems, developmental and environmentally-induced PCD execution can be influenced by the ubiquitous eukaryotic cellular process of autophagy [24–27]. Autophagy is a process that sequesters and reutilizes intracellular contents mediated by double-membrane vesicles called autophagosomes which shuttle contents to the lysosome (animals) or vacuole (plants) for degradation and recycling [25, 28, 29]. The autophagy phenomenon in plants can play a dual role in promoting or inhibiting PCD making the ability to distinguish the exact function of autophagy in plant PCD difficult [28, 30–32]. To study the core machinery behind autophagosome formation and fusion to lytic bodies, autophagy-related genes (ATGs) across eukaryotic models have been investigated and characterized. For example, in animal systems, the Atg5-Atg12-Atg16 E3-like complex tethers the Atg8 to the originating phagophore by conjugation to Atg8-PE before the phagophore completes closure [33–38]. In plants, the impact of Atg16 on autophagy regulation is not determined compared to Atg8 which has been studied more extensively [39].

The autophagy process is important to characterize across plant diversity due to its capability of promoting or inhibiting PCD in plant stress response or development although its precise place in the PCD pathway is uncertain [25]. Knockdown experiments across different plant tissues have shown individual Atg proteins play a direct role in autophagy and PCD performance during development [25]. RNA interference (RNAi) of *Atg5* and *Atg6* suppresses vacuolar cell death and promotes uncontrolled necrosis in suspensor cells of developing *Picea abies* embryos [40]. Knockout of core autophagy gene transcripts suppresses hypersensitive cell death in *Arabidopsis* and *Nicotiana benthamiana* leaves under pathogen infection [30, 41–45]. The distinct roles of autophagosome formation and vacuolar breakdown in PCD initiation, execution, and inhibition may be different in plant cells destined for death or survival [25]. The lace plant PCD model system is a uniquely positioned system to further elucidate the mechanisms of plant autophagy. The NPCD/PCD cell ‘gradient’ of lace plant window leaves provides a tractable platform to investigate autophagy promotion or inhibition in a cell-specific setting [29] similar to the *P*. *abies* embryo-suspensor system [5, 9, 25] to better understand the involvement of autophagy in plant PCD.

Dauphinee et al., (2019) [29] utilized commercially available autophagy modulators rapamycin, wortmannin, and concanamycin A (ConA) to treat lace plants to determine if autophagic activity helped promote or inhibited lace plant PCD. Their findings showed that the promotion or inhibition of autophagy did not affect the induction of developmental PCD, evidenced by no significant change in the number of perforations formed in mature leaves. However, the promotion of autophagy significantly decreased the rate of cell death events in late-stage PCD cells [29] while autophagy inhibitors had the opposite effect. These results indicated that autophagy is active in PCD and NPCD cells during lace plant leaf development but mainly promotes cell survival. However, we speculate that there is a dual role for autophagy occurring in each of these cell types.

The only Atg protein investigated in lace plant development is Atg8 which is detectable in NPCD and PCD cells by using immunostaining but not differentially expressed between cell types and leaf stages [29, 46]. Additionally, a transcriptomic analysis of each developmental stage of lace plant leaves revealed that transcripts for *Atg16* and *Atg18a* are highly up-regulated in pre-perforation leaves and window leaves compared to mature leaves and imperforate leaves [46] while other detectable *Atgs* genes remained unchanged throughout leaf development. Atg16 has been implicated in the regulation of autophagosome formation in animals and yeast but its specific role in plant autophagy and PCD is not confirmed [39, 47].

The goal of this study is to characterize Atg16 levels during lace plant leaf development and to further elucidate the role of plant autophagy and its involvement in lace plant leaf development and PCD. We use previously optimized treatment protocols of known autophagy modulators rapamycin, wortmannin, or ConA on lace plants to compare Atg16 levels, anthocyanin concentration, and the formation of perforations in leaves at different stages of development. As a working hypothesis, we propose that autophagy activity promotion by modulation leads to a significant increase in Atg16 levels and a decrease in NPCD cell anthocyanin accumulation which ultimately affects PCD in terms of the number of perforations formed in lace plant leaves.

## Materials and methods

### Lace plant propagation and treatments

Whole lace plant cultures were aseptically propagated as described in Gunawardena et al. (2020) [48]. Newly obtained corms of lace plants [*A*. *madagascariensis* (Mirbel) H. Bruggen] were obtained from The PlantGuy (Alberta, Canada) and cultured in GA-7 Magenta boxes, embedded fully in solid MS media [100 ml of 1.5% plant tissue culture agar (w/v, Phytotech Laboratories) in liquid MS (3% sucrose (w/v), 0.01% Myo-inositol (w/v), 0.215% MS basal salts (w/v) Phytotech Laboratories), 0.0025% thiamine-HCl (v/v), pH 5.7] and then submerged in 150 ml of liquid MS. Whole plant cultures were grown at 24°C and exposed to 12 h light: 12 h dark cycles with levels of 125 μmol m^-2^ s^-1^ daylight deluxe fluorescent light bulbs (Philips). Cultured plants were only selected for pharmacological experimentation after 30 d of growth and the production of 3 perforated mature leaves to control for variation in plant growth.

Plants selected for pharmacological experiments were treated for 1 week with either (i) 5 μM rapamycin (Enzo Scientific, BML-275), (ii) 5 μM wortmannin (Cayman Chemical, 10010591), or (iii) 1 μM ConA (Santa Cruz Biotechnology, sc-202111). Control plants received an equal volume of dimethyl sulfoxide DMSO (<0.1% v/v; BioShop Canada, DMS666). A minimum of six replicates were performed for each group (four groups total).

### RNA extraction from lace plant leaf stages

RNA was extracted from individual leaf stages from plants grown for 30 d under normal conditions to measure the accumulation of Atg16 mRNA during lace plant leaf development. Mid-rib free leaf lamina tissue samples were washed with distilled water, blotted dry, and flash-frozen. RNA was extracted from 40 mg of frozen tissue from one of each imperforate, pre-perforation, window, or mature stage leaves from 3 different whole-plant cultures and processed as per instructions for the ReliaPrep RNA Kit (Promega). RNA samples were treated with DNAse I (Thermo Fisher). Eluted RNA was quantified using a Nanodrop spectrophotometer (Thermo Fischer) by measuring absorbance at 260 nm.

RNA was extracted from window and mature leaf stages from treated and control plants in the same manner as described above. Leaf lamina tissue samples were obtained from 3 different whole-plant cultures treated with either rapamycin, wortmannin, ConA, or DMSO (control).

### Quantification of Atg16 transcripts in lace plant leaves

Transcripts for lace plant *Atg16* were used to verify the synthesis results of Atg16 protein RNA sequencing (RNA-Seq) by calculating the relative fold-change in gene expression of samples using the 2^-ΔΔCT^ method by qRT-PCR [46]. Equal amounts of RNA (0.1 μg) from treated and control leaf stages were used as a template for cDNA conversion. Single-strand cDNA was synthesized using SuperScript®III First-Strand Synthesis System for qRT-PCR (Invitrogen, Canada) and oligo dT_20_ following the manufacturer’s instructions. All sample replicate cDNA conversions were performed without reverse transcriptase to verify for contamination of genomic DNA as a control.

qRT-PCR was conducted on a Rotor-Gene RG-3000 system (Corbett Research, Sydney, NSW, Australia) using 0.5 μl cDNA as a template and 0.4 mmol l^−1^ primers for *Atg16* or *α-tubulin* (S1 Table) under the following conditions: 5 min at 94°C, 35 cycles of 30 s at 94°C, 30 s at 54°C and 1 min at 72°C, followed by 72°C. A QuantiFast® SYBER® Green PCR Kit (Qiagen, Canada) was used for the qPCR procedure. Melt curve analysis was completed using Rotor-Gene 6 Software and experiments with at least 90% efficiency were used for analysis (Corbett Research, Australia). The experiment was performed in triplicate using three biological replicates of imperforate, pre-perforation, window, and mature stage leaves; each preparation was analyzed in duplicate. cDNA copy numbers for *Atg16* were determined from a standard curve of Ct values (R^2^ > 0.99) and normalized against the lace plant *α-tubulin* isoform as described and verified in Rowarth et al. (2021) [46].

Transcript copy numbers for lace plant *Atg16* and *α-tubulin* were determined by qPCR to measure the accumulation of mRNA in window leaves after treatment with autophagy modulators. Equal amounts of RNA (0.1 μg) from treated and control window and mature stage leaf stages were used as a template for cDNA conversion and qRT-PCR was conducted with RNA from biological replicates of the window and mature leaves from plants treated with DMSO control, rapamycin, wortmannin or ConA. The experiment was performed in triplicate using lace plant leaf stages from three individual plants.

### Detecting Atg16 protein levels in lace plant leaves

Protein extraction and western blotting protein detection were carried out as described in (Rowarth et al., 2020) [48] to measure Atg16 protein amounts in lace plant leaf stages. One of each imperforate, pre-perforation, window, and mature leaves from normally grown plants was harvested. Leaf stages were individually excised from the midrib, rinsed gently with distilled water, blotted dry on filter paper, and then flash-frozen. Frozen leaf tissues were homogenized individually on ice 1:1 (w:v) in a 1% HALT™ protease inhibitor cocktail (Fisher Scientific, #78430) diluted in PIPES buffer solution (100 mM PIPES, 1 mM MgCl_2_, 1 mM EGTA, pH 6.8). Homogenates were centrifuged at 16 000 *g* at 4°C for 15 min, the supernatants were removed, and total protein was quantitated using a Bradford assay. Protein samples were diluted 1:1 with 2× Laemmli sample buffer (Bio-Rad) containing 5% β-mercaptoethanol (v/v) and boiled for 5 min. A 10-μg aliquot of each protein sample and a Precision Plus Protein Standard (Bio-Rad) were loaded onto an 8–16% SDS–polyacrylamide Mini-PROTEAN TGX pre-cast gel (Bio-Rad) and resolved at a constant 250 V for 30 min in ice-cold running buffer [0.1% SDS (v/v), 25 mM Tris, and 192 mM glycine, pH 8.3]. Proteins were transferred at a constant 100 V for 30 min onto 0.2 μm nitrocellulose membranes (Bio-Rad) in ice-cold transfer buffer [20% methanol (v/v), 25 mM Tris, and 192 mM glycine, pH 8.3]. Membranes were stained in Ponceau S stain [0.1% (w/v) Ponceau (Sigma), 5% (v/v) acetic acid] for 2 min and then washed in TBS-T (Tris-buffered saline–Tween: 10 mM Tris, 140 mM NaCl, and 0.1% Tween-20, pH 7.4) for 10 s and imaged by the scanner before being blocked in 5% (w/v) low-fat milk powder in TBS-T for 1 h at room temperature. Membranes were then incubated at 4°C overnight in primary rabbit anti-Atg16 antibody (anti-Atg16, Agrisera, #AS19 4280) diluted 1:10 000 in 4% milk in TBS-T and washed four times in TBS-T for 1, 2, 3, and 4 min, respectively. Membranes were incubated at 1:20 000 in TBS-T for 30 min in goat anti-rabbit: horseradish peroxidase (HRP) secondary antibody (Agrisera, #AS10 667) and washed as before followed by an additional 3 min wash in TBS (10 mM Tris, 140 mM NaCl, pH 7.4). After membrane washing, antibody-reactive protein bands were visualized using Clarity ECL Reagent (Bio-Rad) and an MF-ChemiBIS 3.2 gel documentation system (DNR Bio-Imaging Systems). Ponceau S-stained protein lanes and immunoreactive band intensities for Atg16 were quantitated with Image Studio Lite Software (Li-Cor Biosciences). Atg16 band intensities were compared between leaf stages. Protein lanes on nitrocellulose stained with Ponceau S served as a loading control. The experiment was performed in triplicate.

Protein extraction for window stage leaves from plants treated with different autophagy modulators was performed as described above. The most recently grown window stage leaf from each of the DMSO control, rapamycin, wortmannin, and ConA-treated plants were harvested, with leaf blades excised from the midrib, blotted dry on filter paper, and then flash-frozen. Frozen leaf tissues were homogenized individually on ice 1:1 (w:v) in a 1% HALT™ protease inhibitor cocktail diluted in PIPES buffer solution. Homogenates were centrifuged at 16 000 *g* at 4°C for 15 min, the supernatants were removed, and total protein was quantitated using a Bradford assay. Protein samples were diluted 1:1 with 2× Laemmli sample buffer containing 5% β-mercaptoethanol (v/v) and boiled for 5 min. A 10-μg aliquot of each protein sample was separated by SDS-PAGE as described above and transferred to 0.2 μm nitrocellulose membranes and probed and imaged with anti-Atg16 antibody as described before. The experiment was performed in triplicate.

### Anti-Atg16 antibody reactivity test

Recombinant lace plant Atg16 protein was cloned and synthesized to test for Anti-Atg16 antibody reactivity and specificity by western blotting. For the production of full length recombinant lace plant *Atg16*, cDNA for the protein was generated by PCR using Platinum *Taq* Polymerase (Invitrogen, Canada), 0.2 mM primers containing NcoI and HindIII restriction enzyme sites (S1 Table) and 0.5 μg of cDNA from lace plant pre-perforation leaves as a template using the PCR reaction: 5 min at 94°C, 30 cycles of 30 s at 94°C, 30 s at 53°C, and 1 min at 72°C, followed by 10 min at 72°C. The cDNA was digested with NcoI and HindIII (New England BioLabs, USA) at 37°C overnight and purified with a Wizard® SV PCR Clean-Up System (Promega). Digested cDNA product was ligated into the PRSET-C His-tagged prokaryotic expression vector (Invitrogen) that had been digested with NcoI and HindIII and the newly recombinant plasmids were then transformed into *E*. *coli* BL21(DE3) pLysS (Invitrogen) for purification. Lace plant Atg16 recombinant protein synthesis was induced with 1 mM isopropyl thio-ß-D-galactoside (IPTG, Thermo Fischer) for 6 h at 37°C and the recombinant Atg16 were purified from cell-free extracts of *E*. *coli* with the MagneHis™ Protein Purification System (Promega).

To test for antibody reactivity, 0.1 μg of recombinant lace plant Atg16 protein, 20 μg of lace plant pre-perforation leaf, 20 μg window stage leaf cell-free extracts, and a Precision Plus Protein Standard were resolved in an 8–16% SDS–polyacrylamide Mini-PROTEAN TGX pre-cast gel (Bio-Rad), transferred to nitrocellulose, blocked, and probed with anti-Atg16 prior to washing and imaging as described above. This protocol was adapted from Rowarth and MacRae (2018) [49].

### Anthocyanin content quantification of treated leaves

Quantification of anthocyanin in leaves harvested from treatment and control conditions was performed as described in Dauphinee et al. (2017) and Rowarth et al. (2020) [16, 48] to test if lace plant leaf anthocyanin concentration is affected by autophagy modulators. Twenty mg of excised leaf tissue from the window and mature leaves from axenic whole-plant treatment conditions (described above) were excised and macerated in 200 μl of formic acid/methanol (5:95, v/v). Samples were incubated on ice in the dark for 50 min, followed by 10 min centrifugation at 10 000 *g*. The supernatant was collected, and absorbance was read at 520 nm using a SmartSpec Plus Spectrophotometer (Bio-Rad). Results were expressed as cyanidin-3-rutinoside equivalents (C3REs), and standard curves of cyanidin-3-rutinoside (anthocyanin) were used to calibrate concentration. A minimum of three replicates were used for each group.

### Image analysis and processing

Leaf images were captured using a Nikon L110 digital camera. Photoshop and Illustrator (Adobe Creative Cloud; Adobe Systems Inc.) were used to prepare images of leaves and western blots for publication. Images of detached leaves had backgrounds removed using Photoshop. A Nikon AZ100 microscope acquired micrographs of window leaf areoles post-treatments. Image processing was consistent for all micrograph figures and included background removal, and adjustments for brightness, contrast, and color balance.

### Statistical analysis and data representation

One-way ANOVA or Two-way ANOVA followed by a Tukey test was used to identify significant differences among means for all treated mature leaf perforation counts, mature leaf lengths, window stage leaf anthocyanin content, leaf *Atg16* transcript copy numbers, and leaf protein Atg16 protein band intensity experimental comparisons. All data are presented as the mean ± SE (α = 0.05). Analyses were carried out using GraphPad Prism 5 software (GraphPad Software Inc.).

## Results

### Anti-Atg16 was immunoreactive with recombinant lace plant Atg16

The anti-Atg16 antibody reacted to purified recombinant lace plant Atg16 alongside pre-perforation and window stage leaf protein extracts all generating bands ~56 kDa in size (S1 Fig).

### Atg16 is up-regulated in pre-perforation and window stage leaves

**Fig 2:**
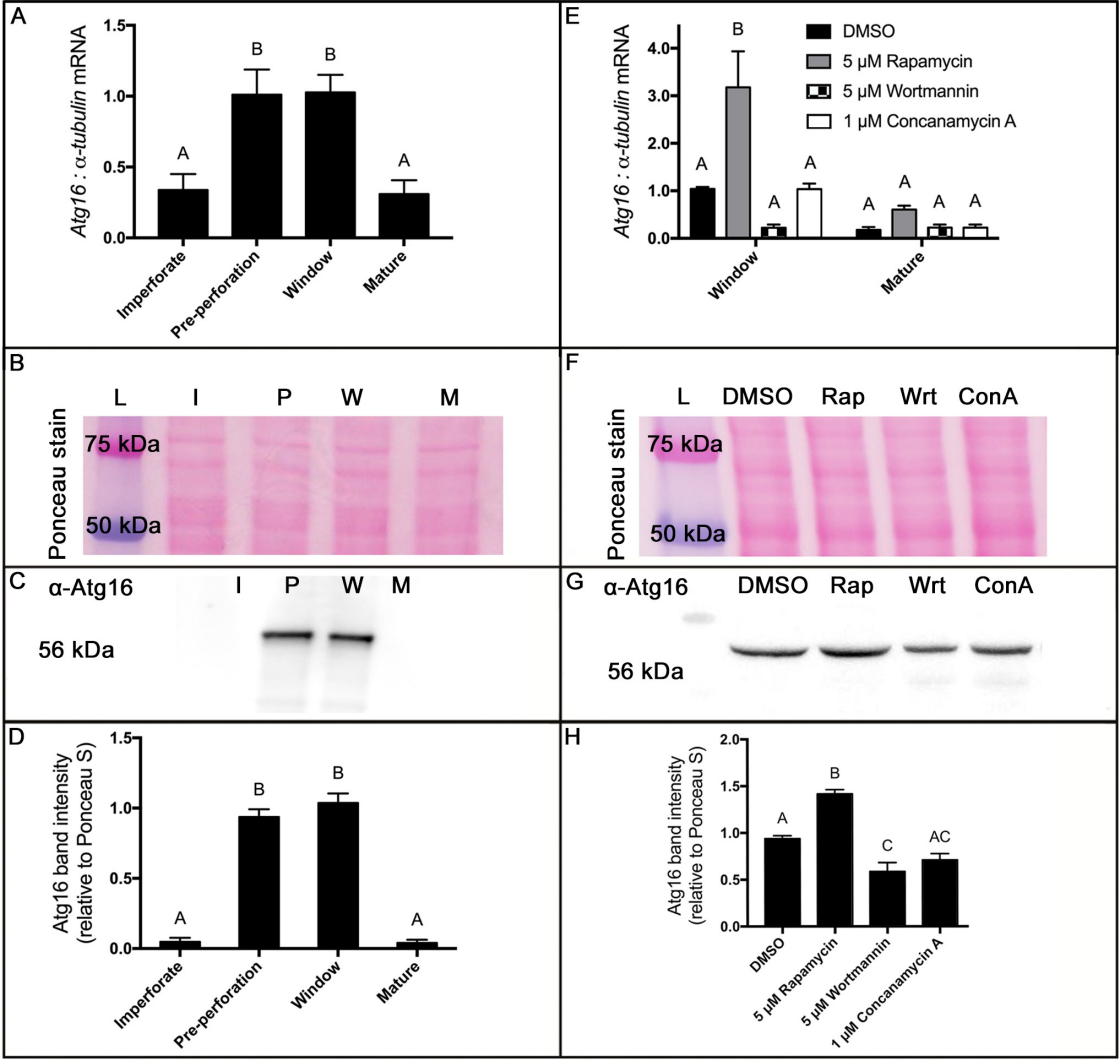
Detection of lace plant Atg16 in lace plant leaves. RNA and protein were extracted and probed for Atg16 from imperforate (I), pre-perforation (P), window (W), or mature (M) lace plant leaf stages (**A**-**D**) or only window and mature leaves from plants treated with either 5 μM rapamycin (Rap), 1 μM wortmannin (Wrt), and 1 μM concanamycin A (ConA) compared to DMSO control (**E**-**H**). (**A** and **E**) The mean levels of *Atg16* mRNA in different lace plant leaf stages were determined by qRT-PCR and normalized to lace plant *α-tubulin* levels. Protein extracts and a molecular protein standard (L) were resolved by SDS–polyacrylamide gels and blotted to nitrocellulose membranes. (**B** and **F**) Membranes and protein lanes were stained with Ponceau-S to serve as loading control before subsequent detection of the presence or absence of ~56 kDa sized Atg16 protein bands with anti-Atg16 antibody (**C** and **G**). (**D** and **H**) Immunoreactive protein bands were quantitated, and the ratio of Atg16 band intensity to the Ponceau lane signal was averaged. The experiments were performed in triplicate. Means not sharing any letter are significantly different. One-way ANOVA (**A**, **D** and **H**), or Two-way ANOVA, Tukey test (**E**, *P* < 0.05; *n* = 3). *Error bars* represent the SE.

*Atg16* mRNA and protein in individual imperforate, pre-perforation, window, and mature leaves were measured to characterize their accumulation across leaf development. Imperforate and mature leaf Atg16 mRNA levels were not significantly different (*P* = 0.9985) and both were significantly lower than pre-perforation and window stage leaves (*P* < 0.05; Fig 2A). Pre-perforation and window leaf *Atg16* mRNA levels were not significantly different (*P* = 0.9997).

Protein detection by western blotting was used to investigate Atg16 protein levels in leaves at different developmental stages (Fig 2B–2D). Imperforate and mature leaf Atg16 protein levels were not significantly different (*P* = 0.9996) and both were significantly lower than pre-perforation and window stage leaves (*P* < 0.0001). Pre-perforation and window leaf Atg16 protein levels were not significantly different (*P* = 0.4996; Fig 2D, normalized to the Ponceau S-stained protein lane signal).

### Autophagy modulators affected Atg16 levels in window stage leaves

The level of *Atg16* mRNA in window leaves from plants treated with rapamycin was significantly higher (approximately 3-fold) than in DMSO controls (*P* = 0.0010; Fig 2E). *Atg16* levels in wortmannin and ConA-treated plants were not significantly different compared to control (*P* > 0.05). No autophagy modulator treatment significantly changed *Atg16* levels in mature leaves compared to control mature leaves or control window leaves (*P* > 0.05).

Protein detection by western blotting was used to investigate if autophagy modulators affected Atg16 protein levels in window stage leaves of treated leaves (Fig 2F–2H). Band intensities of Atg16 protein were normalized to the first replicate of window stage leaves from control plants and quantified for window stage leaves from plants treated with rapamycin, wortmannin, and ConA (Fig 2H). Window stage leaves of rapamycin-treated plants had significantly higher Atg16 levels (normalized to Ponceau S-stained protein lanes) compared to window stage control leaves (*P* = 0.0047). Atg16 levels were significantly lower in window stage leaves of wortmannin-treated plants than controls (*P* = 0.0320). Levels of Atg16 were not significantly different between window stage leaves of ConA-treated plants and wortmannin-treated leaves (*P* = 0.7201).

### Autophagy modulators affected mature leaf perforations

**Fig 3:**
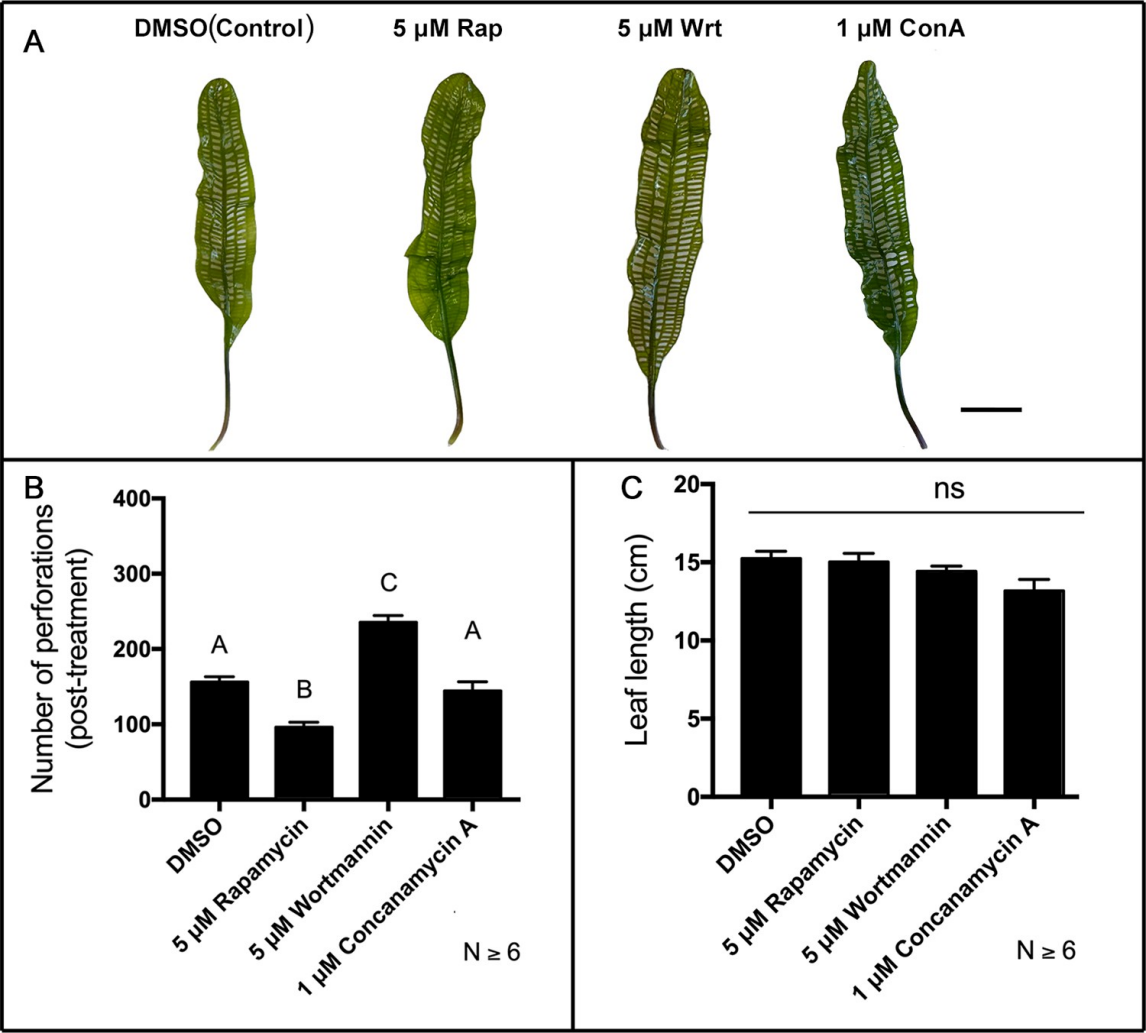
Effects of autophagy modulation treatment on mature leaf perforations. (**A**) Representative leaves from plants treated with 5 μM rapamycin (Rap), 1 μM wortmannin (Wrt), and 1 μM concanamycin A (ConA) compared to DMSO control. (**B**) Mean number of perforations formed in mature leaves post-treatment. (**C**) Mean leaf lengths of mature leaves post-treatment. (**B**-**C**) Means not sharing any letter are significantly different. One-way ANOVA, Tukey test (*ns* = non-significant, *P* > 0.05, *n* ≥ 6). *Error bars* represent the SE. *Scale bars* = 2 cm.

The number of perforations formed in the most recently developed mature leaves was recorded after one week of treatment with either DMSO control, rapamycin, wortmannin, or ConA (Fig 3). Mature leaves from DMSO control plants produced 156.19 ± 6.61 perforations. Mature leaves from rapamycin plants produced significantly fewer perforations (94.18 ± 6.43 perforations, *P* = 0.0002) compared to the control. Conversely, weeklong treatment with wortmannin produced mature leaves with significantly more perforations compared to the control (235.56 ± 8.71, *P* < 0.0001). Weeklong treatment with ConA did not significantly alter the number of perforations formed (Fig 3B; 144.56 ± 11.41, *P* = 0.9447) compared to control. There was no significant difference in leaf length between treatments and control (Fig 3C; *P* > 0.05).

### Autophagy modulators affected window stage leaf anthocyanin levels

**Fig 4:**
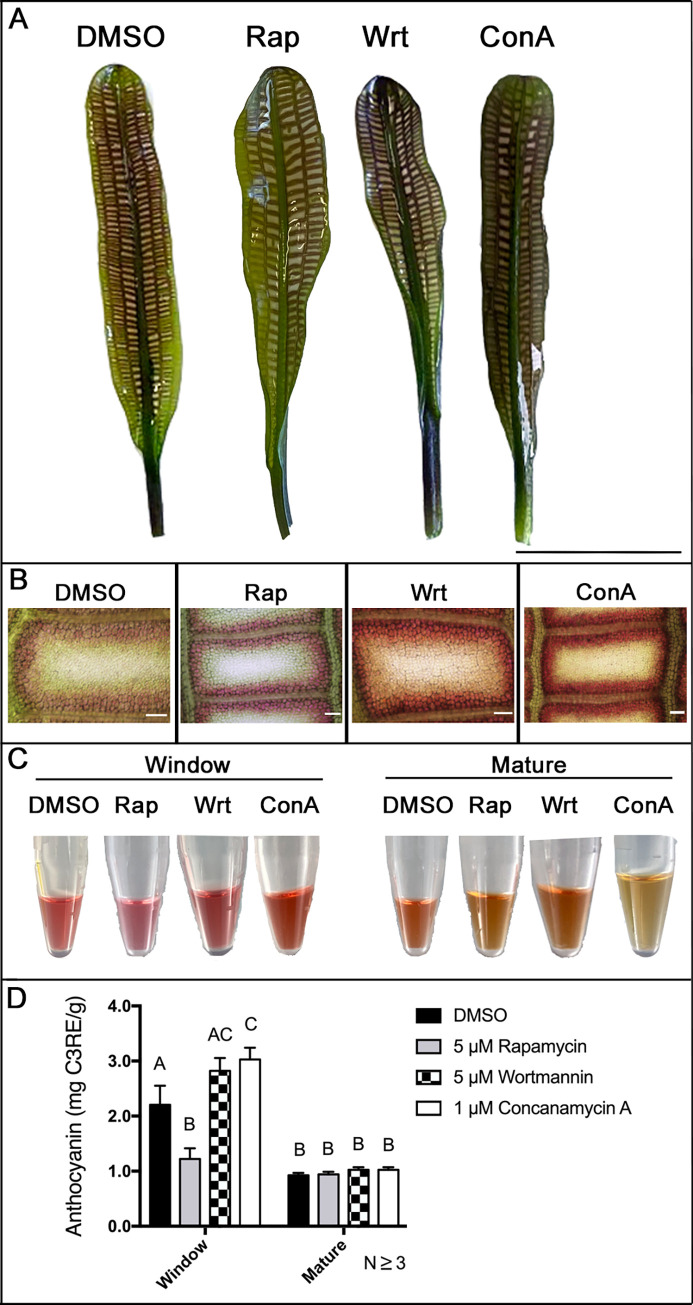
Anthocyanin concentration of autophagy modulator treated window or mature stage leaves. (**A**) Window stage leaves from plants treated with either DMSO (control), 5 μM rapamycin (Rap), 5 μM wortmannin (Wrt), or 1 μM concanamycin A (ConA) and (**B**) corresponding micrographs of representative areoles. (**C**) Anthocyanin extracted from the window and mature leaves from treated plants and (**D**) absorbance values of window and mature leaves from plants treated with either DMSO (control), 5 μM rapamycin (Rap), 1 μM wortmannin (Wrt), or 1 μM concanamycin A (ConA). Mean anthocyanin concentrations were plotted as standard equivalents of cyaniding-3-rutinoside (C3REs). Means not sharing any individual letters are significantly different. Two-way ANOVA, Tukey test. (*ns* = non-significant, *P* > 0.05, *n* ≥ 3). *Error bars* represent the SE. *Scale bars*: A = 2 cm; B = 50 μm.

The anthocyanin content of window and mature stage leaves from plants treated with autophagy modulators was quantified and compared to DMSO controls (Fig 4). Window stage leaves from control plants had a mean anthocyanin content of 2.5 mg ± 0.07 C3RE/g which was significantly higher than the anthocyanin content from control mature leaves (Fig 4D; 0.98 ± 0.062 C3RE/g, *P* < 0.0001). Rapamycin treatment resulted in significantly lower anthocyanin in window stage leaves (1.05 ± 0.25 C3RE/g, *P* < 0.0001) than control window stage leaves but did not significantly differ from control mature leaves (*P* = 0.9993). The concentration of anthocyanin in window stage leaves from wortmannin-treated plants did not differ significantly from control window leaves (3.13 ± 0.21 C3RE/g, *P* = 0.3690) but was significantly higher than rapamycin-treated window leaves (*P* < 0.0001). Window stage leaves from ConA-treated plants produced significantly higher amounts of anthocyanin (3.32 ± 0.07 C3RE/g, *P* = 0.0018) compared to control window leaves but did not differ significantly from wortmannin-treated leaves (*P* > 0.05). The amount of anthocyanin in mature leaves regardless of treatment was not significantly different from control mature leaves (*P* = 0.9543).

## Discussion

Previous investigations using the lace plant model have elucidated the effects of autophagy modulators on Atg8 levels, NPCD cells, and PCD cells during leaf development (Dauphinee et al., 2019) [29]. Even though there was no direct involvement of Atg8 protein in the PCD process of perforation formation, autophagy played a key role in leaf development by mediating proper cell death rates in PCD cells and survival in NPCD cells. However, there is still a need for investigating the effects of other autophagy-related proteins, Atg16 levels, and autophagy modulators on lace plant leaf anthocyanin content and perforation formation during development has yet to be determined. Since autophagy is implicated in developmental PCD in other systems like xylem formation in *Arabidopsis* [50], suspensor deletion in Norway spruce embryos [25], differentiation of proximal root cap cells [51, 52] and anthocyanin modulation [53, 54] we set out to further elucidate this mechanism by investigating the involvement of another key protein, Atg16, and the effects of autophagy on lace plant leaf anthocyanin and development.

### Atg16 was developmentally regulated and levels affected by autophagy modulators

We measured Atg16 levels by qPCR and western blotting to confirm its synthesis during lace plant leaf development. Both Atg16 mRNA and protein levels were significantly higher in pre-perforation and window stage leaves compared to mature and imperforate leaves. These levels are correlated with early stages of lace plant leaf development when PCD and anthocyanin accumulation is active (Fig 2A and 2D) and are also consistent with the RNA-Seq data revealed in Rowarth et al., (2021) [46]. Pre-perforation and window stage leaves have been shown to up-regulate transcripts encoded for genes involved in autophagy promotion such as *Atg16*, *Atg18a*, and *SNF1-related protein kinase KIN10* [46, 55] suggesting an increased need for autophagosome formation and cell maintenance during early lace plant developmental PCD.

Lace plants were treated with autophagy modulators rapamycin, wortmannin, or ConA to confirm the effects autophagy activity has on Atg16 levels in window stage leaves and leaf development in general. Rapamycin caused Atg16 levels to significantly increase in window leaves, and wortmannin had the opposite effect (Fig 2E and 2H). AZD-8055 (another TOR suppressor) treatment has previously shown that the promotion of autophagosome-like bodies and Atg8 puncta in lace plant cells, similar to rapamycin treatment (56), indicating a promotion of autophagic activity [29]. Our results show that Atg16 levels are correlated with autophagic activity in window stage leaves, but future experiments with other autophagy promoters like AZD-8055 or nutrient deprivation should be performed to confirm autophagic effects on Atg protein levels. ConA treatment did not affect Atg16 levels (Fig 2E and 2H) but was shown to increase autophagic body build up in lace plants [56]. This suggests that prevention of autophagic vacuolar breakdown either does not provide feedback to affect or only causes subtle changes to the Atg16 biosynthesis in lace plants.

Knockdown experiments in *Dictyostelium discoideum* have shown that Atg16 is needed for autophagosome formation and is important for crosstalk between autophagy and the ubiquitin proteosome system [57]. *Atg16* is detected in early roots and leaves of *Oryza sativa* seedlings and its one homolog is slightly inducible by salt, cold, desiccation, and dark treatment [58]. The lace plant Atg proteins along with Atg16 likely work in concert to ensure that proper balance of leaf growth and nutrient requirements are met during early leaf development and down-regulated in maturity and imperforate leaves when Atg16 is no longer required. Homologs of *Atg16* have been sequenced and characterized in several other plant species but it is not determined if Atg16 plays a critical requirement in activating plant autophagy [39, 58, 59]. The advancement of functional-transformational studies showing how plants develop without Atg16 will help elucidate its role in plant PCD.

### Autophagy modulators affected lace plant leaf anthocyanin levels and PCD

Plants treated with rapamycin generated window stage leaves with significantly lower anthocyanin concentration (Fig 4D) and led to fewer perforations formed in mature leaves (Fig 3B). This result is similar to other lace plant leaf pharmacological treatments with exogenous antioxidants [16, 48], auxin transport inhibitor NPA [60], and Hsp70 inhibitor PES-Cl [48], which all lowered anthocyanin production, superoxide generation in PCD cells, and total perforations formed. Autophagy, Hsp70, and antioxidants are known to mitigate ROS generation by degrading deleterious protein aggregates and ROS-generating organelles through chlorophagy and mitophagy [61–64]. Higher activity of autophagy and suppression of ROS generation in PCD cells as well as lower anthocyanin concentration in NPCD cells may be the lead cause of lower cell death rates under rapamycin treatment [29]. These results, taken together with previous pharmacological experiments, indicate that departure from the basal imbalance of the ROS-anthocyanin gradient threshold within areoles leads to irregular or inhibited lace plant PCD. We consistently have observed that ROS accumulation in PCD cells is correlated to anthocyanin accumulation in NPCD cells [16, 48, 60]. Our observations that rapamycin inhibited the formation of perforations (this study) and cell death rates [29] suggest inhibition of ROS accumulation in PCD cells as well. Therefore, using the cell death assay and measuring the number of perforations formed is a suitable proxy to determine ROS (and ultimately PCD) is inhibited when autophagy is promoted by rapamycin.

ConA-treated leaves raised anthocyanin levels similar to wortmannin but generated mature leaves with fewer perforations compared to wortmannin. These results show that both of these autophagy inhibitors increased the likelihood of stress in NPCD cells, visible as a higher concentration of anthocyanin (Fig 4B) but differentially affected the number of perforations formed (Fig 3B), a measure of the extent of PCD. Wortmannin treated lace plant leaves increase the number of vesicles containing organelles in NPCD cells, inhibits Atg8 synthesis and reduce autophagosome-like bodies in lace plant leaves [29, 56] indicating the inhibiton of autophagic activity.

The exact mechanism that causes wortmannin and ConA to generate lace plant leaves with similar raised anthocyanin levels but with a different number of perforations is unknown. We speculate these different effects are caused from the different ways these modulators affect autophagic activity. ConA increases the number vacuolar aggregates and Atg8 puncta accumulated in the vacuole of *P*. *abies* suspensor cells [25] and lace plant leaf cells [56]. ConA inhibits vacuolar H^+^-ATPases [65] preventing the degradation of accumulated autophagic bodies in the vacuole by raising vacuolar pH and preventing the activity of hydrolases [66]. It is likely that late-phase autophagy inhibition by ConA causes a compounding of stress in NPCD cells such as loss of hydrolase activity and recycled nutrients which leads to the higher accumulation of anthoycanins and then increased cell death rates in PCD cells [29]. The up-stream autophagy inhibition by wortmannin may cause even higher rates of cell death in PCD cells. While the down-stream inhibition of autophagy coupled with the possible pleiotropic effects of ConA may still cause enough stress to raise anthocyanin levels but not enough to significantly affect the formation of perforations.

We cannot rule out the limitations of the autophagy modulators used to study autophagy in the lace plant model system. Plants are known to be less sensitive to rapamycin which can affect metabolism and growth. However, it has been shown that separately the lace plant [29] and *Arabidopsis* in liquid MS medium can partially recover this sensitivity [67]. Since the lace plant is an aquatic plant, this might represent a path for aquatic plant species to be more sensitive to rapamycin treatment. The phosphatidylinositol 3-kinase (PI3K) inhibitors wortmannin and 3-methyladenine (3-MA) can affect endosomal and vacuolar trafficking while 3-MA has been documented to both promote and inhibit autophagy in a time dependent manner [68]. 3-MA has been previously tested in lace plant experiments but showed a poor response intracellularly compared to wortmannin and ConA and thus was not used in these experiments (unpublished thesis work; [69]). As well, autophagy modulation has the capabilities to modify gene expression of other plant and algae *Atgs* [25, 70, 71]. Future experiments should be used to separate which Atgs along with Atg16 are influenced specifically by developmentally-, treatment- and nutrient deprived-induced autophagy.

Dauphinee et al., (2019) [29] previously reported that week-long autophagy modulator treatments did not significantly change the number of mature leaf perforations. However, the previous investigation used a different *A*. *madagascariensis* cultivar that naturally produced thinner mature leaves with fewer rows of perforations, which we no longer have access to. The present cultivar used in this study provides a larger sample size of potential perforations per leaf to survey post-treatment which may explain the differences in results between the two investigations.

How plant autophagy affects anthocyanin biosynthesis and accumulation is not fully understood. Autophagy is at least partially involved in anthocyanin shuttling to vacuoles in plants [53, 72, 73]. Mutant *Arabidopsis* seedlings for *Atg5*, *Atg9*, and *Atg10* accumulate fewer anthocyanin vacuolar inclusions (AVIs) and corresponding changes in anthocyanin profiles compared to wild-type [53]. Anthocyanin transport to the vacuole is believed to be controlled by novel selective autophagy mechanisms [72]. Atgs and other autophagosome interacting proteins like Hsp70, NBR1 and Exo70B1 may help selectively guide anthocyanin cargo to the vacuole for accumulation as shown in *Arabidopsis* [74–77]. Future experiments investigating anthocyanin profiles between NPCD and PCD cells are ongoing to elucidate and resolve the roles of different anthocyanins in cell differentiation and survival.

## Conclusions and future work

Cell type-specific approaches are important to resolve the roles of autophagy in different contexts like stress or development [51, 78], making the lace plant PCD gradient a suitable model to study the relationship between autophagy and plant PCD. Taken together, our results show that higher levels of Atg16 in lace plant window leaves were induced by rapamycin and were inversely correlated to anthocyanin concentration and the number of perforations formed in mature leaves; the opposite effect was observed with wortmannin. The link between autophagic activity, monitored through Atg16 levels, and anthocyanin levels as well as perforation formation, points to a role for autophagy in mediating the amplitude of lace plant PCD.

More recent studies like Feng et al., (2022) [51] have shown that PCD and corpse clearance dependency on autophagy is cell-type specific during *Arabidopsis* lateral root cap and columella development. Possibly, a similar scenario is in place in lace plant leaf morphogenesis where cell-specific differences between NPCD and PCD cells occur. Future lace plant experiments should utilize live-cell imaging and lace plant *Atg* knockout lines to measure autophagic activity more accurately within the 48 h window of cell death [79].

**Fig 5:**
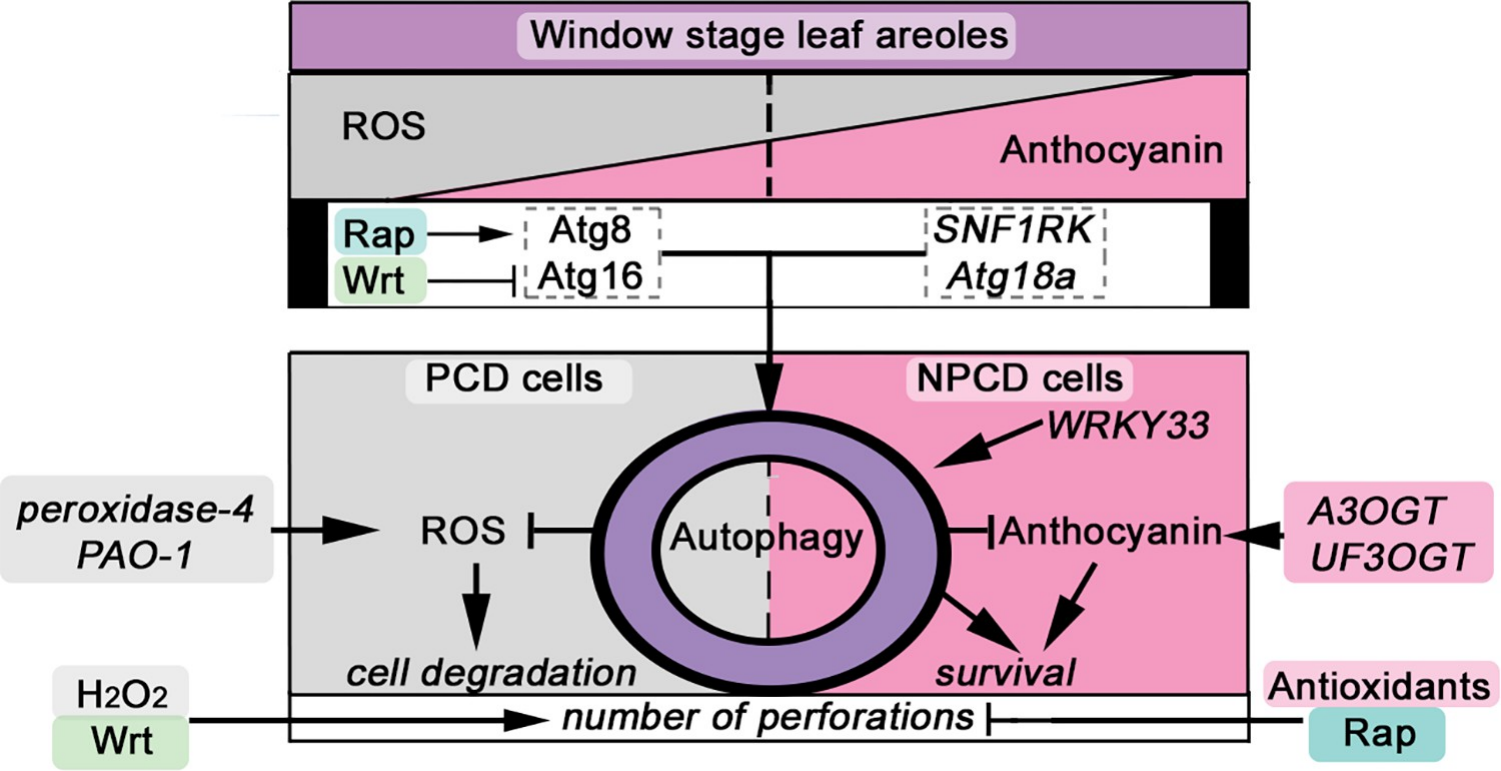
Diagram of potential interactions between lace plant PCD, ROS, anthocyanin, autophagy and pertinent genes. An unknown induction signal(s) during window leaf development initiates the differentiation of PCD from NPCD cells within the areole, visible by an imbalance between reactive oxygen species (ROS) and anthocyanin [16]. RNA-Seq analysis has shown that window leaves up-regulate genes such as Atg18a and SNF1RK that promote autophagy [46]. During window stage of leaf development Atg8 [29] and Atg16 protein levels are high and can be manipulated by autophagy modulators [this study, 56]. PCD cells up-regulate ROS [16] and ROS generating genes peroxidase-4 and primary amine oxidase-1 (*PAO-1*) while NPCD cells up-regulate anthocyanidin (*A3OGT*) and UDP flavonoid-3-O-glucosyltransferases (*UF3OGT*) that promote the production of anthocyanins and a *WRKY33* transcription factor that promotes autophagy [46]. Wortmannin (Wrt) and exogenous ROS in the form of H_2_O_2_ have similar effects on window stage leaves possibly accentuating cell death and the ROS-anthocyanin gradient imbalance [this study, 16]. On the other hand, rapamycin (Rap) and exogenous antioxidants inhibit anthocyanin accumulation [this study, 16, 48], cell death [29] and perforation formation [This study, 48]. When the ROS-anthocyanin imbalance threshold is not reached this consistently leads to inhibited lace plant PCD [16, 48, 60]. Under normal conditions autophagy can mediate ROS [80] and anthocyanin levels (this study). How autophagic activity, the ROS-anthocyanin ‘gradient’ and associated molecular targets all mediate PCD and intracellular communication between PCD and NPCD cells requires further investigation.

We summarize the involvements of autophagy, Atg16, anthocyanin, ROS, and a variety of other genes in lace plant leaf development (Fig 5). This shows that disruptions in the autophagic machinery can affect the potency of PCD response in lace plant leaf development. The results from this investigation infer a role for autophagy and other up-regulated *Atg* genes in window stage leaves to help optimize anthocyanin accumulation and promote survival in NPCD cells and mediate a timely cell death in PCD cells.

### Contribution to the field statement

Autophagy has been reported to play pro-cell death and pro-survival roles in mediating programmed cell death (PCD). Recent plant PCD studies have highlighted the need for the resolution of autophagy’s role in developmental plant PCD in a cell and time-specific manner across plant models. *Aponogeton madagascariensis*, commonly known as the lace plant, utilizes developmental PCD to form perforations in its mature leaves. The first visible sign of PCD is the disappearance of anthocyanins central of areoles, the laminar tissue bounded by veins. This creates a gradient of the red pigment from anthocyanin and PCD progression as well as a visible scheme to spatiotemporally separate cells destined for death and cells destined to survive. It is less well understood if autophagy promotes or inhibits lace plant PCD and anthocyanin accumulation during leaf development. Atg16 is a core machinery protein behind animal autophagic activity, but its role in plant PCD is unknown. We chose to probe for its synthesis during lace plant leaf development to measure autophagy. The results here show that autophagy promotion in lace plants by treatment with rapamycin inhibited anthocyanin accumulation and PCD, generating mature leaves with fewer perforations. This indicates a role for autophagy in mediating proper anthocyanin levels and PCD establishment during lace plant leaf development and building our understanding of how autophagy affects plant anthocyanins.

## Supporting information

S1 Table Primers used for PCR experiments. (XLSX)

S1 Fig Anti-Atg16 reactivity. Protein extract from lace plant leaves and recombinant *AmAtg16* were resolved in SDS polyacrylamide gels, transferred to nitrocellulose, and probed with anti-Atg16 antibody. Lane 1, protein standard ladder; 2, 0.1 μg of recombinant *AmAtg16* protein; 3, 20 μg of protein extract from lace plant pre-perforation leaf stage; 4, 20 μg of protein extract from lace plant window leaf stage. *Black arrows* indicate targeted and reactive ~56 kDa bands detected in protein lanes.
(PDF)

S2 Fig Raw image of Atg16 leaf developmental regulation western blot. Protein extract from lace plant imperforate, pre-perforation, window, and mature stage leaves were resolved in SDS polyacrylamide gels, transferred to nitrocellulose, and probed with anti-Atg16 antibody. Lane 1, protein standard ladder; 2, 10 μg of imperforate stage leaf protein; 3, 10 μg of protein extract pre-perforation leaf stage; 4, 10 μg of protein extract from window stage leaf; 5, 10 μg of protein extract from mature stage leaf. Sample lanes not used in final image due to loading error are annotated with an ‘X’.
(PDF)

S3 Fig Raw image of Atg16 window leaf autophagy modulator response western blot. Protein extract from lace plant window leaves treated with different autophagy modulators were resolved in SDS polyacrylamide gels, transferred to nitrocellulose, and probed with anti-Atg16 antibody. Lane 1, protein standard ladder; 2, 10 μg of DMSO control window stage leaf protein; 3, 10 μg of rapamycin-treated window stage leaf protein; 4, 10 μg of wortmannin-treated window stage protein leaf protein leaf stage; 5, 10 μg of ConA-treated window stage leaf protein. Sample lanes not used in final image due to loading error are annotated with an ‘X’.
(PDF)

S4 Fig Raw image of Anti-Atg16 reactivity western blot. Protein extract from lace plant leaves and recombinant *AmAtg16* were resolved in SDS polyacrylamide gels, transferred to nitrocellulose, and probed with anti-Atg16 antibody. Lane 1, protein standard ladder; 2, 0.1 μg of recombinant *AmAtg16* protein; 3, 20 μg of protein extract from lace plant pre-perforation leaf stage; 4, 20 μg of protein extract from lace plant window leaf stage. Sample lanes not used in final image due to loading error are annotated with an ‘X’.
(PDF)
