# Supplementary figures and images for: The role of Atg16 in autophagy, anthocyanin biosynthesis, and programmed cell death in leaves of the lace plant (Aponogeton madagascariensis)
Source: PLoS One. 2023 Feb 16;18(2):e0281668. doi: 10.1371/journal.pone.0281668 (PMC9934333; doi:10.1371/journal.pone.0281668)

**1****2****3****4****56 kDa-**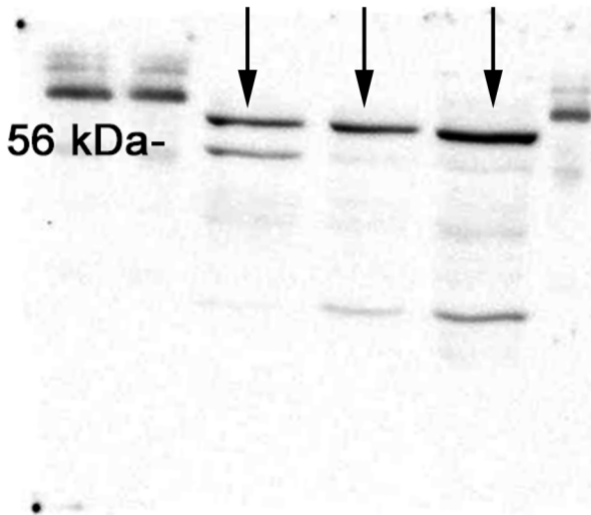

Supplement: S1 Fig — Protein extract from lace plant leaves and recombinant AmAtg16 were resolved in SDS polyacrylamide gels, transferred to nitrocellulose, and probed with anti-Atg16 antibody. Lane 1, protein standard ladder; 2, 0.1 μg of recombinant AmAtg16 protein; 3, 20 μg of protein extract from lace plant pre-perforation leaf stage; 4, 20 μg of protein extract from lace plant window leaf stage. Black arrows indicate targeted and reactive ~56 kDa bands detected in protein lanes. (PDF) [file pone.0281668.s002.pdf]

X X X X X X X 1 2 3 4 5

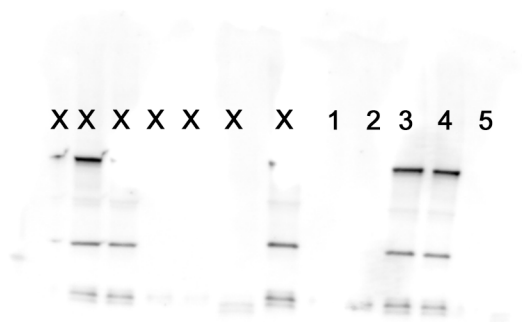

Supplement: S2 Fig — Protein extract from lace plant imperforate, pre-perforation, window, and mature stage leaves were resolved in SDS polyacrylamide gels, transferred to nitrocellulose, and probed with anti-Atg16 antibody. Lane 1, protein standard ladder; 2, 10 μg of imperforate stage leaf protein; 3, 10 μg of protein extract pre-perforation leaf stage; 4, 10 μg of protein extract from window stage leaf; 5, 10 μg of protein extract from mature stage leaf. Sample lanes not used in final image due to loading error are annotated with an ‘X’. (PDF) [file pone.0281668.s003.pdf]

1 2 3 4 5 X X X

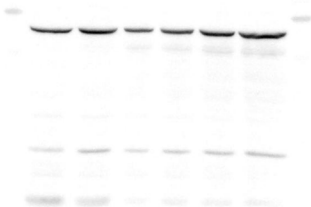

Supplement: S3 Fig — Protein extract from lace plant window leaves treated with different autophagy modulators were resolved in SDS polyacrylamide gels, transferred to nitrocellulose, and probed with anti-Atg16 antibody. Lane 1, protein standard ladder; 2, 10 μg of DMSO control window stage leaf protein; 3, 10 μg of rapamycin-treated window stage leaf protein; 4, 10 μg of wortmannin-treated window stage protein leaf protein leaf stage; 5, 10 μg of ConA-treated window stage leaf protein. Sample lanes not used in final image due to loading error are annotated with an ‘X’. (PDF) [file pone.0281668.s004.pdf]

X 1 2 3 4 1 X X X

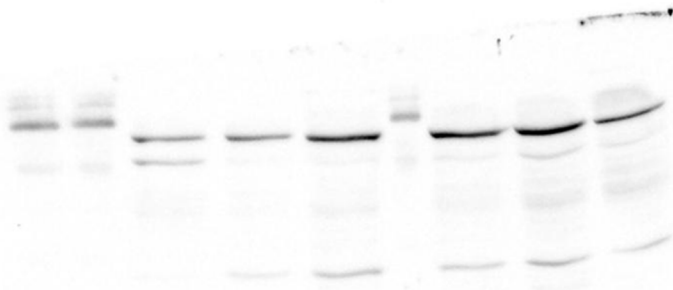

Supplement: S4 Fig — Protein extract from lace plant leaves and recombinant AmAtg16 were resolved in SDS polyacrylamide gels, transferred to nitrocellulose, and probed with anti-Atg16 antibody. Lane 1, protein standard ladder; 2, 0.1 μg of recombinant AmAtg16 protein; 3, 20 μg of protein extract from lace plant pre-perforation leaf stage; 4, 20 μg of protein extract from lace plant window leaf stage. Sample lanes not used in final image due to loading error are annotated with an ‘X’. (PDF) [file pone.0281668.s005.pdf]
